# Diaqua­bis­(4-carb­oxy-2-propyl-1*H*-imidazole-5-carboxyl­ato-κ^2^ *N* ^3^,*O* ^4^)copper(II) *N*,*N*-dimethyl­formamide disolvate

**DOI:** 10.1107/S1600536810025249

**Published:** 2010-07-07

**Authors:** Lan-Zhen He, Shi-Jie Li, Wen-Dong Song, Dong-Liang Miao

**Affiliations:** a College of Science, Guang Dong Ocean University, Zhanjiang 524088, People’s Republic of China; b College of Food Science and Technology, Guang Dong Ocean University, Zhanjiang 524088, People’s Republic of China

## Abstract

In the title complex, [Cu(C_8_H_9_N_2_O_4_)_2_(H_2_O)_2_]·2C_3_H_7_NO, the Cu^II^ ion, lying on an inversion center, is six-coordinated in a slightly distorted octa­hedral geometry. Two N atoms and two O atoms from two H_2_pimda (H_3_pimda is 2-propyl-1*H*-4,5-dicarb­oxy­lic acid) ligands are in the equatorial plane. The axial positions are occupied by two O atoms from two water mol­ecules. A two-dimensional supra­molecular network parallel to (001) is constructed by N—H⋯O and O—H⋯O hydrogen bonds. An intra­molecular O—H⋯O hydrogen bond is also observed.

## Related literature

For the potential uses and diverse structural types of metal complexes with imidazole-4,5-dicarb­oxy­lic acid, see: Li *et al.* (2006 ▶); Liu *et al.* (2004 ▶); Sun *et al.* (2005 ▶); Zou *et al.* (2006 ▶).
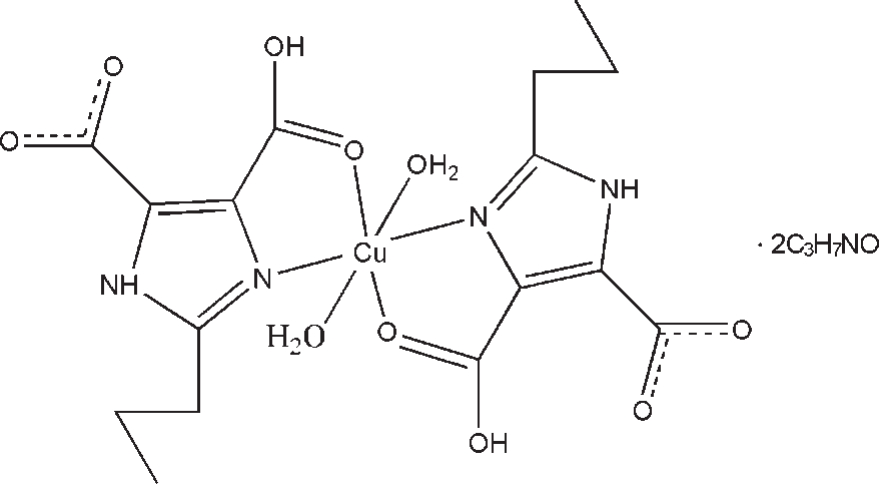


## Experimental

### 

#### Crystal data

[Cu(C_8_H_9_N_2_O_4_)_2_(H_2_O)_2_]·2C_3_H_7_NO
*M* *_r_* = 640.11
Triclinic, 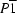

*a* = 7.2831 (8) Å
*b* = 9.250 (1) Å
*c* = 11.3329 (13) Å
α = 75.264 (1)°
β = 87.305 (2)°
γ = 68.416 (1)°
*V* = 685.68 (13) Å^3^
*Z* = 1
Mo *K*α radiation
μ = 0.87 mm^−1^
*T* = 298 K
0.32 × 0.21 × 0.19 mm

#### Data collection

Bruker SMART 1000 CCD diffractometer
Absorption correction: multi-scan (*SADABS*; Sheldrick, 1996 ▶) *T*_min_ = 0.768, *T*_max_ = 0.852
3603 measured reflections
2385 independent reflections
2011 reflections with *I* > 2σ(*I*)
*R*_int_ = 0.017

#### Refinement

*R*[*F* ^2^ > 2σ(*F* ^2^)] = 0.039
*wR*(*F* ^2^) = 0.097
*S* = 1.06
2385 reflections
187 parameters
H-atom parameters constrained
Δρ_max_ = 0.38 e Å^−3^
Δρ_min_ = −0.29 e Å^−3^

### 

Data collection: *SMART* (Bruker, 2007 ▶); cell refinement: *SAINT* (Bruker, 2007 ▶); data reduction: *SAINT*; program(s) used to solve structure: *SHELXS97* (Sheldrick, 2008 ▶); program(s) used to refine structure: *SHELXL97* (Sheldrick, 2008 ▶); molecular graphics: *SHELXTL* (Sheldrick, 2008 ▶); software used to prepare material for publication: *SHELXTL*.

## Supplementary Material

Crystal structure: contains datablocks I, global. DOI: 10.1107/S1600536810025249/hy2320sup1.cif

Structure factors: contains datablocks I. DOI: 10.1107/S1600536810025249/hy2320Isup2.hkl

Additional supplementary materials:  crystallographic information; 3D view; checkCIF report

## Figures and Tables

**Table 1:** Hydrogen-bond geometry (Å, °)

*D*—H⋯*A*	*D*—H	H⋯*A*	*D*⋯*A*	*D*—H⋯*A*
N2—H2⋯O6^i^	0.86	1.83	2.679 (3)	167
O2—H2*A*⋯O3	0.82	1.67	2.494 (3)	177
O5—H5*A*⋯O4^ii^	0.85	1.91	2.755 (3)	172
O5—H5*B*⋯O4^iii^	0.85	2.07	2.906 (3)	167

Symmetry codes: (i) 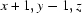; (ii) 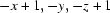; (iii) 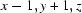.

**Fig. 1.**
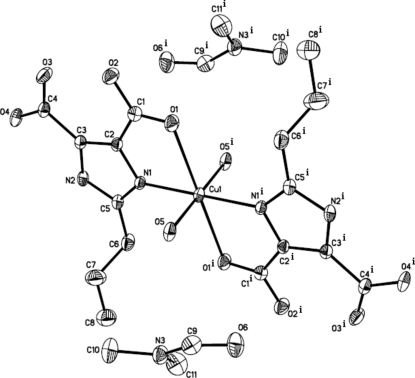
Molecular structure of the title compound. Displacement ellipsoids are shown at the 30% probability level. H atoms are omitted for clarity. [Symmetry code: (i) 1-x, 1-y, 1-z.]

**Fig. 2.**
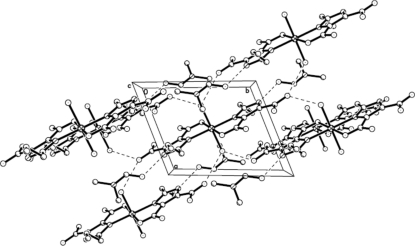
A view of the two-dimensional network constructed by O—H···O and N—H···O hydrogen bonding interactions. H atoms are omitted for clarity.

## supplementary crystallographic information

### Comment

Design and synthesis of metal-organic complexes *via* deliberate selection of metal ions and organic ligands have been one of the most attractive subjects due to their fascinating structures and potential applications in many field. It is well known that ligands containing N and O atoms which are highly accessible to metal ions are good candidates for the design and synthesis. For example, imidazole-4,5-dicarboxylic acid (H_3_idc) containing N and O coordination sites can be deprotonated to form (H_2_idc)^-^, (Hidc)^2-^ and (idc)^3-^ anions at different pH values. H_3_idc has been widely used to react with metal salts to obtain a series of metal-organic frameworks with different structures and useful properties (Li *et al.*, 2006; Liu *et al.*, 2004; Sun *et al.*, 2005; Zou *et al.*, 2006). Therefore, we chose 2-propyl-imidazole-4,5-dicarboxylic acid (H_3_pimda) as ligand for the synthesis of fascinating structures and we report a new Cu^II^ complex here.

As illustrated in Fig. 1, the asymmetric unit of the title complex comprises one H_2_pimda ligand, one Cu^II^ ion lying on an inversion center, one coordinated water molecule and one solvent DMF molecule. The Cu^II^ ion is six-coordinated in a slightly distorted octahedral geometry, formed by two N atoms and two O atoms from two H_2_pimda ligands in the equatorial plane. The Cu—O bond length with the value of 2.458 (2) Å is somewhat longer than the Cu—N bond with the value of 1.987 (2) Å. The axial positions are occupied by two O atoms from two water molecules [Cu—O = 2.020 (2) Å]. The H_2_pimda ligand adopts a bidentate mode to chelate the metal atom through one imidazole N atom and one O atom from the protonated carboxyl group. The other carboxyl group is deprotonated, indicated by a difference of the bond lengths. The two imidazole rings are coplanar. The DMF molecules are linked to the H_2_pimda ligand *via* N—H···O hydrogen bonds. The two-dimensional supramolecular network is stabilized by N—H···O and O—H···O hydrogen bonds (Fig. 2, Table 1).

### Experimental

A mixture of Cu(NO_3_)_2_ (0.5 mmol, 0.05 g) and 2-propyl-1*H*-imidazole-4,5-dicarboxylic acid (0.5 mmol, 0.99 g) in 15 ml of DMF solution was sealed in an autoclave equipped with a Teflon liner (20 ml) and then heated at 433 K for 4 d. Blue crystals were obtained by slow evaporation of the solvent at room temperature.

### Refinement

C- and N-bound H atoms were placed at calculated positions and were treated as riding on the parent atoms, with C—H = 0.93 (CH), 0.97 (CH_2_) and 0.96 (CH_3_) Å, N—H = 0.86 Å, and with *U*_iso_(H) = 1.2(1.5 for methyl)*U*_eq_(C, N). H atoms of the water molecule and hydroxyl group were located in a difference map and were allowed to ride on the parent atom, with O—H = 0.85 and 0.82 Å and *U*_iso_(H) = 1.2(1.5 for hydroxyl)*U*_eq_(O).

### Crystal data

**Table:**

[Cu(C_8_H_9_N_2_O_4_)_2_(H_2_O)_2_]·2C_3_H_7_NO	*Z* = 1
*M**_r_* = 640.11	*F*(000) = 335
Triclinic, *P*1	*D*_x_ = 1.550 Mg m^−^^3^
Hall symbol: -P 1	Mo *K*α radiation, λ = 0.71073 Å
*a* = 7.2831 (8) Å	Cell parameters from 1702 reflections
*b* = 9.250 (1) Å	θ = 2.5–25.9°
*c* = 11.3329 (13) Å	µ = 0.87 mm^−^^1^
α = 75.264 (1)°	*T* = 298 K
β = 87.305 (2)°	Cubic, blue
γ = 68.416 (1)°	0.32 × 0.21 × 0.19 mm
*V* = 685.68 (13) Å^3^	

### Data collection

**Table:**

Bruker SMART 1000 CCD diffractometer	2385 independent reflections
Radiation source: fine-focus sealed tube	2011 reflections with *I* > 2σ(*I*)
graphite	*R*_int_ = 0.017
φ and ω scans	θ_max_ = 25.0°, θ_min_ = 1.9°
Absorption correction: multi-scan (*SADABS*; Sheldrick, 1996)	*h* = −8→7
*T*_min_ = 0.768, *T*_max_ = 0.852	*k* = −10→10
3603 measured reflections	*l* = −10→13

### Refinement

**Table:**

Refinement on *F*^2^	Primary atom site location: structure-invariant direct methods
Least-squares matrix: full	Secondary atom site location: difference Fourier map
*R*[*F*^2^ > 2σ(*F*^2^)] = 0.039	Hydrogen site location: inferred from neighbouring sites
*wR*(*F*^2^) = 0.097	H-atom parameters constrained
*S* = 1.06	*w* = 1/[σ^2^(*F*_o_^2^) + (0.0414*P*)^2^ + 0.548*P*] where *P* = (*F*_o_^2^ + 2*F*_c_^2^)/3
2385 reflections	(Δ/σ)_max_ < 0.001
187 parameters	Δρ_max_ = 0.38 e Å^−^^3^
0 restraints	Δρ_min_ = −0.29 e Å^−^^3^

### Fractional atomic coordinates and isotropic or equivalent isotropic displacement parameters (Å^2^)

**Table:**

	*x*	*y*	*z*	*U*_iso_*/*U*_eq_	
Cu1	0.5000	0.5000	0.5000	0.02632 (17)	
N1	0.6271 (3)	0.2621 (3)	0.5343 (2)	0.0251 (5)	
N2	0.7981 (3)	0.0045 (3)	0.5992 (2)	0.0295 (6)	
H2	0.8707	−0.0862	0.6461	0.035*	
N3	0.1268 (4)	0.4896 (3)	0.8656 (3)	0.0434 (7)	
O1	0.4276 (3)	0.4348 (2)	0.31460 (19)	0.0387 (5)	
O2	0.4952 (3)	0.2191 (3)	0.24467 (19)	0.0422 (6)	
H2A	0.5573	0.1221	0.2687	0.063*	
O3	0.6933 (4)	−0.0740 (3)	0.3193 (2)	0.0442 (6)	
O4	0.8653 (3)	−0.2477 (2)	0.4863 (2)	0.0398 (5)	
O5	0.2402 (3)	0.4858 (2)	0.56026 (19)	0.0353 (5)	
H5A	0.2186	0.4061	0.5485	0.042*	
H5B	0.1412	0.5701	0.5293	0.042*	
O6	0.0414 (4)	0.7502 (3)	0.7641 (2)	0.0568 (7)	
C1	0.5080 (4)	0.2896 (3)	0.3284 (3)	0.0305 (7)	
C2	0.6222 (4)	0.1888 (3)	0.4434 (3)	0.0255 (6)	
C3	0.7286 (4)	0.0262 (3)	0.4834 (3)	0.0265 (6)	
C4	0.7665 (4)	−0.1092 (3)	0.4254 (3)	0.0305 (7)	
C5	0.7356 (4)	0.1463 (3)	0.6284 (3)	0.0289 (7)	
C6	0.7787 (5)	0.1646 (4)	0.7491 (3)	0.0414 (8)	
H6A	0.7328	0.2782	0.7460	0.050*	
H6B	0.9208	0.1199	0.7659	0.050*	
C7	0.6827 (7)	0.0825 (6)	0.8528 (4)	0.0694 (12)	
H7A	0.5414	0.1240	0.8336	0.083*	
H7B	0.7328	−0.0316	0.8573	0.083*	
C8	0.7160 (7)	0.1037 (5)	0.9754 (3)	0.0659 (12)	
H8A	0.8439	0.0288	1.0098	0.099*	
H8B	0.6159	0.0845	1.0282	0.099*	
H8C	0.7097	0.2114	0.9668	0.099*	
C9	0.0181 (5)	0.6215 (4)	0.7857 (3)	0.0451 (8)	
H9	−0.0840	0.6167	0.7424	0.054*	
C10	0.0974 (8)	0.3399 (5)	0.8779 (4)	0.0779 (14)	
H10A	−0.0212	0.3605	0.8322	0.117*	
H10B	0.0856	0.2932	0.9625	0.117*	
H10C	0.2082	0.2669	0.8471	0.117*	
C11	0.2925 (6)	0.4885 (5)	0.9321 (4)	0.0625 (11)	
H11A	0.4116	0.4480	0.8910	0.094*	
H11B	0.3049	0.4207	1.0134	0.094*	
H11C	0.2709	0.5959	0.9361	0.094*	

### Atomic displacement parameters (Å^2^)

**Table:**

	*U*^11^	*U*^22^	*U*^33^	*U*^12^	*U*^13^	*U*^23^
Cu1	0.0281 (3)	0.0167 (3)	0.0329 (3)	−0.0064 (2)	−0.0004 (2)	−0.0065 (2)
N1	0.0284 (13)	0.0187 (11)	0.0282 (13)	−0.0084 (10)	0.0010 (10)	−0.0066 (10)
N2	0.0300 (14)	0.0166 (11)	0.0365 (14)	−0.0047 (10)	−0.0039 (11)	−0.0025 (10)
N3	0.0490 (18)	0.0311 (14)	0.0453 (17)	−0.0112 (13)	−0.0017 (13)	−0.0060 (12)
O1	0.0464 (14)	0.0227 (11)	0.0391 (13)	−0.0053 (10)	−0.0048 (10)	−0.0045 (9)
O2	0.0540 (15)	0.0332 (12)	0.0363 (13)	−0.0100 (11)	−0.0097 (10)	−0.0105 (10)
O3	0.0579 (16)	0.0319 (12)	0.0443 (14)	−0.0110 (11)	−0.0031 (12)	−0.0194 (10)
O4	0.0397 (13)	0.0185 (11)	0.0580 (15)	−0.0045 (9)	−0.0025 (11)	−0.0125 (10)
O5	0.0289 (11)	0.0218 (10)	0.0565 (14)	−0.0094 (9)	0.0037 (10)	−0.0123 (9)
O6	0.0589 (17)	0.0316 (13)	0.0649 (17)	−0.0080 (12)	−0.0174 (13)	0.0038 (12)
C1	0.0282 (16)	0.0297 (16)	0.0334 (17)	−0.0103 (13)	0.0012 (13)	−0.0082 (13)
C2	0.0263 (15)	0.0228 (14)	0.0309 (16)	−0.0122 (12)	0.0038 (12)	−0.0086 (12)
C3	0.0237 (15)	0.0227 (14)	0.0340 (17)	−0.0091 (12)	0.0046 (12)	−0.0083 (12)
C4	0.0256 (16)	0.0236 (15)	0.0445 (19)	−0.0096 (13)	0.0060 (13)	−0.0128 (14)
C5	0.0304 (16)	0.0222 (14)	0.0340 (17)	−0.0100 (12)	−0.0013 (13)	−0.0060 (12)
C6	0.053 (2)	0.0267 (16)	0.0416 (19)	−0.0119 (15)	−0.0131 (16)	−0.0051 (14)
C7	0.085 (3)	0.094 (3)	0.052 (3)	−0.050 (3)	0.019 (2)	−0.035 (2)
C8	0.079 (3)	0.067 (3)	0.051 (2)	−0.024 (2)	0.006 (2)	−0.018 (2)
C9	0.0362 (19)	0.047 (2)	0.048 (2)	−0.0092 (16)	−0.0039 (16)	−0.0138 (17)
C10	0.105 (4)	0.041 (2)	0.094 (3)	−0.033 (2)	0.015 (3)	−0.019 (2)
C11	0.050 (2)	0.058 (2)	0.062 (3)	−0.0083 (19)	−0.0161 (19)	0.001 (2)

### Geometric parameters (Å, °)

**Table:**

Cu1—N1^i^	1.987 (2)	C1—C2	1.475 (4)
Cu1—N1	1.987 (2)	C2—C3	1.377 (4)
Cu1—O5^i^	2.020 (2)	C3—C4	1.491 (4)
Cu1—O5	2.020 (2)	C5—C6	1.481 (4)
Cu1—O1	2.458 (2)	C6—C7	1.519 (5)
N1—C5	1.336 (3)	C6—H6A	0.9700
N1—C2	1.378 (3)	C6—H6B	0.9700
N2—C5	1.344 (3)	C7—C8	1.494 (5)
N2—C3	1.368 (4)	C7—H7A	0.9700
N2—H2	0.8600	C7—H7B	0.9700
N3—C9	1.315 (4)	C8—H8A	0.9600
N3—C11	1.448 (4)	C8—H8B	0.9600
N3—C10	1.449 (4)	C8—H8C	0.9600
O1—C1	1.222 (3)	C9—H9	0.9300
O2—C1	1.305 (3)	C10—H10A	0.9600
O2—H2A	0.8200	C10—H10B	0.9600
O3—C4	1.253 (4)	C10—H10C	0.9600
O4—C4	1.247 (3)	C11—H11A	0.9600
O5—H5A	0.8499	C11—H11B	0.9600
O5—H5B	0.8500	C11—H11C	0.9600
O6—C9	1.226 (4)		
			
N1^i^—Cu1—N1	180.00 (6)	N1—C5—N2	109.4 (2)
N1^i^—Cu1—O5^i^	91.57 (9)	N1—C5—C6	127.0 (3)
N1—Cu1—O5^i^	88.44 (9)	N2—C5—C6	123.6 (3)
N1^i^—Cu1—O5	88.43 (9)	C5—C6—C7	113.2 (3)
N1—Cu1—O5	91.56 (9)	C5—C6—H6A	108.9
O5^i^—Cu1—O5	180.0	C7—C6—H6A	108.9
N1^i^—Cu1—O1	104.94 (8)	C5—C6—H6B	108.9
N1—Cu1—O1	75.06 (8)	C7—C6—H6B	108.9
O5^i^—Cu1—O1	92.58 (8)	H6A—C6—H6B	107.7
O5—Cu1—O1	87.42 (8)	C8—C7—C6	114.9 (3)
C5—N1—C2	106.6 (2)	C8—C7—H7A	108.6
C5—N1—Cu1	134.39 (19)	C6—C7—H7A	108.6
C2—N1—Cu1	118.83 (18)	C8—C7—H7B	108.6
C5—N2—C3	109.7 (2)	C6—C7—H7B	108.6
C5—N2—H2	125.2	H7A—C7—H7B	107.5
C3—N2—H2	125.2	C7—C8—H8A	109.5
C9—N3—C11	119.9 (3)	C7—C8—H8B	109.5
C9—N3—C10	120.6 (3)	H8A—C8—H8B	109.5
C11—N3—C10	119.1 (3)	C7—C8—H8C	109.5
C1—O1—Cu1	108.15 (18)	H8A—C8—H8C	109.5
C1—O2—H2A	109.5	H8B—C8—H8C	109.5
Cu1—O5—H5A	114.3	O6—C9—N3	124.8 (3)
Cu1—O5—H5B	113.0	O6—C9—H9	117.6
H5A—O5—H5B	107.6	N3—C9—H9	117.6
O1—C1—O2	122.2 (3)	N3—C10—H10A	109.5
O1—C1—C2	119.7 (3)	N3—C10—H10B	109.5
O2—C1—C2	118.1 (2)	H10A—C10—H10B	109.5
C3—C2—N1	109.4 (2)	N3—C10—H10C	109.5
C3—C2—C1	132.5 (3)	H10A—C10—H10C	109.5
N1—C2—C1	118.1 (2)	H10B—C10—H10C	109.5
N2—C3—C2	104.9 (2)	N3—C11—H11A	109.5
N2—C3—C4	122.9 (2)	N3—C11—H11B	109.5
C2—C3—C4	132.2 (3)	H11A—C11—H11B	109.5
O4—C4—O3	125.3 (3)	N3—C11—H11C	109.5
O4—C4—C3	117.8 (3)	H11A—C11—H11C	109.5
O3—C4—C3	116.9 (3)	H11B—C11—H11C	109.5
			
O5^i^—Cu1—N1—C5	85.3 (3)	C5—N2—C3—C4	−177.9 (3)
O5—Cu1—N1—C5	−94.7 (3)	N1—C2—C3—N2	−0.5 (3)
O1—Cu1—N1—C5	178.4 (3)	C1—C2—C3—N2	−178.9 (3)
O5^i^—Cu1—N1—C2	−89.3 (2)	N1—C2—C3—C4	177.8 (3)
O5—Cu1—N1—C2	90.7 (2)	C1—C2—C3—C4	−0.7 (5)
O1—Cu1—N1—C2	3.76 (19)	N2—C3—C4—O4	0.3 (4)
N1^i^—Cu1—O1—C1	177.7 (2)	C2—C3—C4—O4	−177.7 (3)
N1—Cu1—O1—C1	−2.3 (2)	N2—C3—C4—O3	179.4 (3)
O5^i^—Cu1—O1—C1	85.4 (2)	C2—C3—C4—O3	1.4 (5)
O5—Cu1—O1—C1	−94.6 (2)	C2—N1—C5—N2	0.1 (3)
Cu1—O1—C1—O2	179.8 (2)	Cu1—N1—C5—N2	−175.00 (19)
Cu1—O1—C1—C2	0.4 (3)	C2—N1—C5—C6	−178.0 (3)
C5—N1—C2—C3	0.3 (3)	Cu1—N1—C5—C6	6.9 (5)
Cu1—N1—C2—C3	176.24 (18)	C3—N2—C5—N1	−0.4 (3)
C5—N1—C2—C1	179.0 (2)	C3—N2—C5—C6	177.8 (3)
Cu1—N1—C2—C1	−5.1 (3)	N1—C5—C6—C7	112.5 (4)
O1—C1—C2—C3	−179.0 (3)	N2—C5—C6—C7	−65.4 (4)
O2—C1—C2—C3	1.7 (5)	C5—C6—C7—C8	−177.7 (3)
O1—C1—C2—N1	2.7 (4)	C11—N3—C9—O6	−2.4 (6)
O2—C1—C2—N1	−176.7 (2)	C10—N3—C9—O6	−175.2 (4)
C5—N2—C3—C2	0.5 (3)		

Symmetry codes: (i) −*x*+1, −*y*+1, −*z*+1.

### Hydrogen-bond geometry (Å, °)

**Table:**

*D*—H···*A*	*D*—H	H···*A*	*D*···*A*	*D*—H···*A*
N2—H2···O6^ii^	0.86	1.83	2.679 (3)	167
O2—H2A···O3	0.82	1.67	2.494 (3)	177
O5—H5A···O4^iii^	0.85	1.91	2.755 (3)	172
O5—H5B···O4^iv^	0.85	2.07	2.906 (3)	167

Symmetry codes: (ii) *x*+1, *y*−1, *z*; (iii) −*x*+1, −*y*, −*z*+1; (iv) *x*−1, *y*+1, *z*.
